# Trends and Factors Associated With Mortality Rates of Leading Causes of Infant Death: A CDC Wide-Ranging Online Data for Epidemiologic Research (CDC WONDER) Database Analysis

**DOI:** 10.7759/cureus.45652

**Published:** 2023-09-20

**Authors:** Okelue E Okobi, Ifreke U Ibanga, Uzoamaka C Egbujo, Thelma O Egbuchua, Kelechukwu P Oranu, Uchechukwu S Oranika

**Affiliations:** 1 Family Medicine, Larkin Community Hospital Palm Springs Campus, Miami, USA; 2 Family Medicine, Medficient Health Systems, Laurel, USA; 3 Family Medicine, Lakeside Medical Center, Belle Glade, USA; 4 Internal Medicine, Thompson General Hospital, Thompson, CAN; 5 Pediatrics, Lagos University Teaching Hospital, Lagos, NGA; 6 Pediatrics and Neonatology, Delta State University Teaching Hospital, Oghara, NGA; 7 Obstetrics and Gynaecology, Kenechukwu Specialist Hospital and Maternity, Enugu, NGA; 8 Family Medicine, University of Alberta Hospital, Edmonton, CAN

**Keywords:** trend analysis, mortality rate, cdc-wonder, infant death, leading cause

## Abstract

Background: Infant mortality is a critical indicator of a nation's healthcare system and social well-being. This study explores trends and factors associated with mortality rates for three leading causes of infant death: congenital malformations, deformations, and chromosomal abnormalities; disorders related to short gestation and low birth weight, not elsewhere classified; and sudden infant death syndrome (SIDS).

Methods: Utilizing the CDC WONDER (CDC Wide-Ranging Online Data for Epidemiologic Research) database, we conducted a retrospective observational analysis of infant mortality rates and associated factors. Data encompassed multiple years, allowing for trend analysis and exploration of influencing variables. Study variables included demographic, maternal, prenatal, and leading cause as factors.

Result: Trends in infant mortality rates varied across causes. The overall mortality rate was 2.69 per 1,000 (p=0.000) people during 2007-2020. The highest rates were observed in 2007 (3.05), 2008 (3.01), and 2009 (2.93) per 1,000 infants. For congenital malformations, deformations, and chromosomal abnormalities, the rate ranged from 1.35 to 1.12 (2007-2020). Gender-based mortality differences were subtle (male rate 2.88 per 1,000 infants, p=0.000; female infants 2.50 per 1,000 infants, p=0.000). The examination of infant mortality trends also explored maternal variables, including maternal age, education, and delivery method. The analysis revealed disparities across variables. Teenage maternal age correlated with higher mortality rates, while maternal education was associated with lower rates. Vaginal delivery (2.61 per 1,000 infants, p=0.199) showed slightly lower rates compared to cesarean section (2.86 per 1,000 infants, p=0.076).

Conclusion: This study utilizes the CDC WONDER database and offers evidence of changing trends in infant mortality rates for the selected causes. Factors such as maternal age (30-34 years and 35-39 years), race/ethnicity (Black or African-American and White), birthplace (in hospital), and mother's education (master's degree) were identified as influencing mortality rates. These findings contribute to informed policymaking and interventions aimed at mitigating infant mortality and improving the well-being of infants and their families. Further research is needed to fully understand the underlying dynamics of these trends and factors.

## Introduction

Infant mortality, which refers to the unfortunate death of a child during the initial year of life, stands as a crucial yardstick for assessing the general health and welfare of a population. The global drive to curtail infant mortality rates has been a central objective of public health campaigns spanning the globe. In order to design effective strategies for intervention and shape policies, it is imperative to grasp the trends and determinants associated with mortality rates linked to the primary causes of infant fatalities [1-2]. In the year 2020 alone, approximately 2.4 million newborns faced mortality worldwide within their first month. This distressing statistic translates to a daily toll of roughly 6,700 newborn deaths, a number that constitutes a staggering 47% of the overall under-five child fatality count, a stark increase from the 40% recorded in 1990. Encouraging progress has materialized since 1990, evident in the reduction of neonatal fatalities from 5 million to 2.4 million by the year 2020 [3].

In 2020, the United States reported the highest infant mortality rate, recording 5.4 deaths for every 1,000 live births, which was significantly greater than Norway's rate, which reported the lowest mortality rate at 1.6 deaths per 1,000 live births [4]. Adding to this disconcerting picture, the maternal mortality rate in the United States for the same year surpassed that of most high-income countries by more than threefold, reaching an alarming 23.8 maternal deaths per 100,000 live births [3-4]. There is a need to meticulously analyze the three primary causes that prominently underlie infant mortality: "congenital malformations, deformations, and chromosomal abnormalities," "disorders related to short gestation and low birth weight, not elsewhere classified," and "sudden infant death syndrome (SIDS)." These factors contribute significantly to the rates of infant mortality and have garnered considerable attention from a spectrum of stakeholders, including researchers, healthcare professionals, and policymakers [5]. The umbrella of "congenital malformations, deformations, and chromosomal abnormalities" encompasses a diverse array of structural anomalies and genetic irregularities that wield substantial influence over infant well-being. Originating during fetal development, these malformations often engender complications within critical organ systems and physiological processes.

The intricate interplay between inherent genetic traits and external environmental factors is underscored by the role they play in the emergence of these abnormalities [1,6-7]. Disorders linked to inadequate gestation and low birth weight embody an intricate interplay of elements entailing maternal well-being, accessibility to prenatal care, and socio-economic circumstances. Infants born with insufficient gestational periods or insufficient birth weights confront a spectrum of health complications encompassing respiratory distress, neurological disorders, and compromised immune systems. These collective factors synergistically expose this demographic of infants to a distinctive susceptibility [8-9]. SIDS, a confounding phenomenon marked by sudden and unexplained infant fatality, introduces a distinctive challenge due to its multifaceted origins. The precise cause of SIDS remains elusive, and it continues to be an area of thorough investigation. Nonetheless, various risk factors have been pinpointed that potentially contribute to its manifestation. These encompass sleeping on the stomach or side, exposure to prenatal or postnatal tobacco smoke, presence of soft bedding in the sleep environment, instances of premature birth or low birth weight, and maternal substance consumption during pregnancy [10-11].

The emotional burden of infant mortality is significant and can have long-lasting effects on families [12]. Parents who lose a child to infant mortality often experience intense feelings of grief and sadness. In some cultures, such as in African and Asian countries, where infant mortality is extremely high, parents may also face social stigma and discrimination. The added burden of other losses and stressors, such as financial strain and employment issues, can further exacerbate the emotional toll of infant mortality. The psychological burden of infant mortality can also be significant for parents and families [13]. Parents who experience the death of a newborn in the neonatal intensive care unit, for example, may experience post-traumatic stress symptoms related to the trauma of the experience. This can lead to psychological distress, likely needing additional support and counseling.

Infant mortality also imposes a significant economic burden on society. In developing countries, the cost of infant mortality is often borne by families who may face significant financial strain. In the United States, the economic burden of infant mortality is mainly due to the cost of medical care and lost productivity. The cost of infant mortality in the United States is estimated to be around $12 billion annually, with $7.4 billion in medical costs and $4.8 billion in lost productivity due to associated disabilities in survivors [14-15]. This economic burden has significant implications for policymakers and society [15]. The long-term impacts of infant mortality on economic growth and development are significant. Studies have shown that infant mortality is associated with lower economic growth and development, as families who experience infant mortality may face significant financial strain and reduced access to education and employment opportunities. This can ripple effect on the broader economy, with reduced productivity and higher healthcare costs [12-15].

The CDC Wide-Ranging Online Data for Epidemiologic Research (CDC WONDER) database stands as a valuable repository for researchers engaged in probing diverse health-related subjects, including infant mortality. Harnessing the extensive dataset within this repository, the present analysis endeavors to unveil trends, correlations, and elements linked to mortality rates concerning the primary causes discussed above [16]. The exploration of these causative factors bears the potential to steer precisely targeted interventions and strategies within public health, designed to drive down infant mortality rates and enhance the overall well-being of neonates and infants.

This research strives to furnish a holistic comprehension of the evolving landscape of infant mortality attributed to these three predominant causes. The inquiry encompasses an array of variables, embracing demographic, maternal, and geographic components. These variables encompass factors like maternal age, educational level, delivery methods, racial backgrounds, and geographical locations. By delving into these variables, the study aims to provide an all-encompassing insight into the intricate interplay between healthcare practices, determinants of societal health, and regional variances that collectively influence the rates of infant mortality.

## Materials and methods

Study design and data source

A retrospective observational design was utilized in this study to analyze trends and factors associated with infant mortality rates for three primary causes: “congenital malformations, deformations, and chromosomal abnormalities”; “disorder related to short gestation and low birth weight”; and “SIDS.” Analyses were carried out between July 20, 2023, and August 11, 2023. The central data source for this analysis was the CDC WONDER database. This database aggregated and granted access to a comprehensive dataset of vital statistics and cause-specific mortality information. All-cause mortality and cause-specific mortality rates for key conditions were examined using the following International Classification of Disease (ICD-10) codes: P07.0 (extremely low birth weight); P07.1 (other low birth weight); P07.2 (extreme immaturity); P07.3 (other preterm infants); Q00-Q99 (congenital malformations, deformations, and chromosomal abnormalities); and R95 (SIDS).

Data collection and variables

The study period encompassed data from 2007 to 2020. The dataset included records of infant deaths attributed to the aforementioned causes, alongside demographic, maternal, and contextual information. The key variables of interest included (a) leading causes of death: These were categorized into congenital malformations, deformations, and chromosomal abnormalities; disorders related to short gestation and low birth weight, not elsewhere classified; and SIDS. (b) Demographic variables: These consisted of the infant's age, gender, and race. (c) Maternal variables: These encompassed maternal age, maternal education, and method of delivery. (d) Geographical variables: These included geographic location (state, census division, and health and human services (HHS region, etc.).

Data analysis

The analysis began with a descriptive overview of the mortality rates for each of the three causes over the study period. Aggregate data for the selected time period (2007-2020) and available patient characteristics were summarized with mortality rates per 1,000 people and the total number of deaths over the years for all patients. Only patients with available data were utilized in each model, and effective sample sizes were included in all tables and figures. All statistical analyses were conducted using Excel version 2019 (Microsoft Corporation, Washington, United States). Trends in mortality rates were presented visually using appropriate graphs and charts. Descriptive analysis: Descriptive statistics were calculated to provide a summary of the study variables. This included measures such as means, medians, standard deviations, and proportions. Trend analysis: Temporal trends in infant mortality rates for each of the three causes were examined using graphical representations to identify significant trends over time. Multiple linear regression was chosen as the statistical method to analyze the relationships between the trends across the study time period and infant mortality rate for leading causes, demographic variables, and maternal factors. The significance of each independent variable was assessed using p-values. The multiple linear regression analysis provided insights into the trends and factors associated with infant mortality rates for leading causes of infant death, contributing to a better understanding of the complex factors affecting infant health and mortality.

Ethical considerations

This study used publicly available, de-identified data from the CDC WONDER database, ensuring the anonymity and privacy of individuals. As the data was collected by the CDC for surveillance purposes, ethical approval and informed consent were not applicable to this secondary analysis.

## Results

Aggregate data were obtained for 149,872 infants (0-1 years of age) who died in the top three leading causes of death between 2007 and 2020 through the CDC WONDER underlying cause of death database. The overall mortality rate per 1,000 people was determined to be 2.69 (p-value=0.000) for the period spanning 2007 to 2020. The highest mortality rates were recorded in 2007 (3.05 per 1,000 infants), 2008 (3.01 per 1,000 infants), and 2009 (2.93 per 1,000 infants), as detailed in Table 1 below.

**Table 1:** Trends in infant mortality rates based on the leading cause of death SIDS: sudden infant death syndrome

Variables	2007	2008	2009	2010	2011	2012	2013	2014	2015	2016	2017	2018	2019	2020	p-value
Total number of death	13,147	12,789	12,117	11,324	11,036	10,856	10,552	10,467	10,501	10,247	9,713	9,515	9,024	8,584	-
Total number of births	4,316,233	4,247,726	4,130,665	3,999,386	3,953,590	3,952,841	3,932,181	3,988,076	3,978,497	3,945,875	3,855,500	3,791,712	3,747,540	3,613,647	-
Overall mortality rate	3.05	3.01	2.93	2.83	2.79	2.75	2.68	2.62	2.64	2.6	2.52	2.51	2.41	2.38	0.000
Congenital malformations	1.35	1.34	1.3	1.28	1.27	1.26	1.22	1.19	1.22	1.22	1.19	1.19	1.15	1.12	0.489
Short gestation and low birth weight	1.13	1.12	1.1	1.04	1.04	1.07	1.07	1.05	1.03	0.99	0.97	0.97	0.92	0.87	0.001
SIDS	0.57	0.55	0.54	0.51	0.48	0.42	0.4	0.39	0.39	0.38	0.35	0.35	0.33	0.38	0.001

Trends in infant mortality rates based on the leading cause of death

Congenital Malformations, Deformations, and Chromosomal Abnormalities

The detailed distribution of mortality rates for each year and each cause of death is provided in Table 1, emphasizing the distinct trends observed for each cause. The study revealed varying trends over the study period for congenital malformations, deformations, and chromosomal abnormalities. The mortality rate per 1,000 people ranged from 1.35 to 1.12 during the years 2007 to 2020 (p-value=0.489). The highest rate was recorded in 2007 (1.35 per 1,000 infants), followed closely by 2008 (1.34 per 1,000 infants). Subsequently, a downward trend was observed, with the lowest rate occurring in 2020 (1.12 per 1,000 infants) (Table 1).

Disorders Related to Short Gestation and Low Birth Weight

The analysis of infant mortality rates due to disorders related to short gestation and low birth weight displayed a declining pattern. The rates per 1,000 people ranged from 1.13 to 0.87 across the years 2007 to 2020 (p-value=0.001). Similar to the trends observed for the leading cause mentioned above, the highest rates occurred in the initial years, notably in 2007 (1.13 per 1,000 infants) and 2008 (1.12). However, a gradual decline was observed thereafter (Table 1).

SIDS

Infant mortality rates associated with SIDS exhibited notable fluctuations over the study period. The rates per 1,000 people ranged from 0.57 to 0.38 from 2007 to 2020 (Table 1). The lowest rate was observed in 2019 (0.33 per 1,000 infants).

The analysis of trends in infant mortality rates based on the three leading causes of death unveiled nuanced variations in rates over the study period. These trends might reflect changes in healthcare practices, interventions, and public health strategies aimed at reducing infant mortality rates associated with these causes.

Trends in infant mortality rates based on demographic variables

The investigation into trends in infant mortality rates took demographic variables, including age, gender, and race, into account. The analysis revealed distinct patterns, as depicted in Table 2. Examination of mortality rates by gender demonstrated subtle differences. While the overall rates were comparable, a slightly higher mortality rate was observed among male infants (2.88 per 1,000 infants; p-value=0.000) compared to female infants (2.50 per 1,000 infants; p-value=0.000), with variations across the years.

**Table 2:** Infant mortality rates based on demographic variables

	Variables	2007	2008	2009	2010	2011	2012	2013	2014	2015	2016	2017	2018	2019	2020	p-value
Gender	Male	3.25	3.24	3.14	3	2.97	2.92	2.87	2.81	2.82	2.82	2.7	2.7	2.57	2.52	0.000
	Female	2.83	2.77	2.72	2.65	2.6	2.56	2.48	2.43	2.45	2.37	2.33	2.31	2.24	2.22	0.000
Race	American Indian or Alaska Native	4.23	3.31	3.84	3.74	3.47	3.84	3.3	3.43	3.68	3.83	3.03	2.89	2.59	-	0.120
	Asian or Pacific Islander	2.17	2.06	1.99	1.92	1.98	1.68	1.76	1.68	1.8	1.61	1.73	1.64	1.6	-	0.081
	Black or African-American	5.53	5.35	5.31	5.04	4.9	4.77	4.65	4.59	4.73	4.67	4.36	4.55	4.35	-	0.000
	White	2.59	2.6	2.5	2.43	2.41	2.4	2.34	2.28	2.26	2.22	2.17	2.13	2.05	-	0.000
Infant age data	Under 1 hour	0.51	0.53	0.52	0.5	0.51	0.5	0.51	0.47	0.47	0.47	0.45	0.45	0.47	0.44	0.006
	1-23 hours	0.97	0.96	0.93	0.91	0.9	0.92	0.9	0.93	0.89	0.79	0.88	0.88	0.85	0.83	0.009
	1-6 days	0.32	0.31	0.31	0.27	0.28	0.3	0.28	0.26	0.29	0.38	0.29	0.28	0.23	0.2	0.056
	7-27 days	0.3	0.27	0.27	0.28	0.27	0.26	0.25	0.24	0.27	0.24	0.23	0.24	0.21	0.22	0.072
	28-364 days	0.95	0.93	0.9	0.86	0.83	0.77	0.74	0.73	0.72	0.73	0.67	0.66	0.65	0.69	0.008

**Figure 1:**
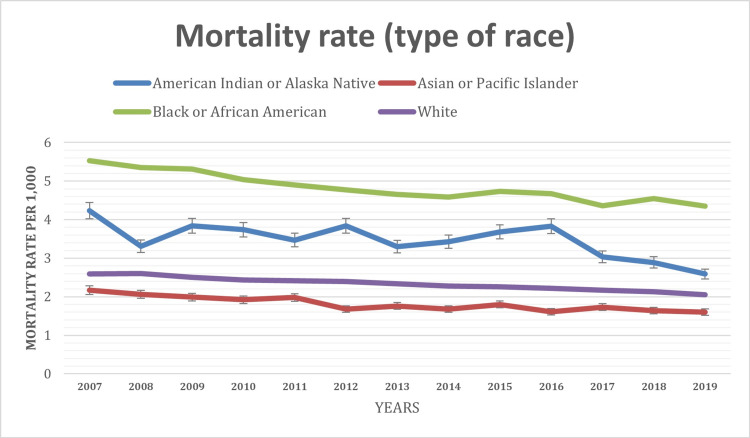
Mortality rate based on type of race (2007-2019)

The analysis of infant mortality rates by race unveiled disparities among racial groups. Rates varied significantly across different racial categories, with the Black race recording the highest mortality (4.83 per 1,000 infants; p-value=0.000), followed by American Indian or Alaska natives (3.48 per 1,000 infants; p-value=0.120), White (2.34 per 1,000 infants; p-value=0.000), and Asian (1.82 per 1,000 infants; p-value=0.081) over the study period (Figure 1).

This underscores the importance of considering racial disparities in understanding infant mortality trends. Furthermore, the analysis demonstrated that infant mortality rates varied significantly across different age groups within the first year of life. The highest mortality rates were consistently observed during the neonatal period at 1-23 hours (0.90 per 1,000 infants; p-value=0.009), followed by 28-364 days (0.77 per 1,000 infants; p-value=0.008), highlighting the vulnerability of newborns.

Trends in infant mortality rates based on maternal variables

The examination of trends in infant mortality rates encompassed an exploration of maternal variables, which included maternal age, education, and method of delivery. Notable trends and patterns that emerged from this analysis are presented in Table 3. The impact of birthplace on infant mortality rates was evident, with hospital deliveries recording lower rates (2.69 per 1,000 infants; p-value=0.056) compared to deliveries outside the hospital (3.09 per 1,000 infants; p-value=0.113). The analysis of infant mortality rates based on the method of delivery showcased differences between infants born through vaginal delivery and those born via cesarean section. Rates were generally lower for infants born through vaginal delivery (2.61 per 1,000 infants; p-value=0.199), while slightly higher rates were observed for infants delivered via cesarean section (2.86 per 1,000 infants; p-value=0.076).

**Table 3:** Infant mortality rates based on maternal variables AA: associate of arts, AS: associate of science, BA: bachelor of arts, AB: artium baccalaureus, BS: bachelor of science, MA: master of arts, MS: master of science, MEng: master of engineering, MEd: master of education, MSW: master of social work, MBA: master of business administration, PhD: doctor of philosophy, EdD: doctor of education, MD: doctor of medicine, DDS: doctor of dental surgery, DVM: doctor of veterinary medicine, LLB: bachelor of laws, JD: juris doctor

	Variables	2007	2008	2009	2010	2011	2012	2013	2014	2015	2016	2017	2018	2019	2020	p-value
Birthplace	In hospital	3.04	3.01	2.92	2.82	2.79	2.74	2.68	2.63	2.63	2.59	2.51	2.5	2.4	2.38	0.056
	Not in hospital	3.83	3.04	3.94	3.7	3.05	2.95	3.14	2.45	3.1	2.82	3.29	2.84	2.79	2.34	0.113
Method of delivery	Vaginal	2.98	2.94	2.86	2.77	2.76	2.69	2.64	2.57	2.55	2.5	2.44	2.42	2.26	2.22	0.199
	Cesarean	3.19	3.15	3.09	2.95	2.85	2.85	2.75	2.73	2.83	2.8	2.68	2.71	2.73	2.7	0.076
Mother age	Under 15 years	5.65	7.29	6.16	6.67	5.79	6.26	6.78	6.5	8.4	4.88	7.3	9.79	7.27	6.8	0.374
	15-19 years	4.27	4.23	3.91	4	3.96	3.75	3.79	3.6	3.51	3.65	3.65	3.59	3.54	3.52	0.015
	20-24 years	3.44	3.36	3.39	3.25	3.17	3.13	3.08	2.98	3.04	3.04	2.95	2.99	2.79	2.85	0.014
	25-29 years	2.71	2.67	2.64	2.49	2.51	2.52	2.49	2.46	2.57	2.47	2.36	2.47	2.37	2.29	0.133
	30-34 years	2.41	2.39	2.35	2.33	2.3	2.27	2.2	2.18	2.15	2.14	2.12	2.04	1.96	1.92	0.040
	35-39 years	2.76	2.82	2.73	2.59	2.57	2.58	2.51	2.49	2.42	2.43	2.38	2.25	2.25	2.23	0.026
	40-44 years	4.44	4.38	3.86	3.92	3.91	3.98	3.81	3.95	3.95	3.62	3.64	3.54	3.44	3.44	0.792
	45-49 years	7.31	5.49	4.92	5.31	6.83	4.89	4.27	5.84	7.22	6.18	3.06	4.87	4.92	4.74	0.152
Mother education	8th grade or less	3.18	3.13	2.74	3.19	3.21	3.21	3.03	2.8	3.14	3.15	3.28	3.22	3.26	3.23	0.926
	9th through 12th grade with no diploma	3.61	3.64	3.71	3.65	3.67	3.69	3.4	3.39	3.65	3.62	3.44	3.47	3.3	3.48	0.091
	Associate degree (AA, AS)	2.28	2.25	2.19	2.25	2.33	2.16	2.15	2.12	2.28	2.24	2.01	2.18	2.09	2	0.818
	Bachelor's degree (BA, AB, BS)	1.78	1.78	1.71	1.65	1.86	1.87	1.9	1.66	1.74	1.61	1.6	1.56	1.5	1.48	0.683
	Master's degree (MA, MS, MEng, MEd, MSW, MBA)	1.72	1.59	1.59	1.53	1.36	1.62	1.53	1.59	1.39	1.4	1.36	1.33	1.44	1.2	0.011
	Doctorate (PhD, EdD) or Professional Degree (MD, DDS, DVM, LLB, JD)	1.3	1.21	1.35	1.58	1.7	1.24	1.39	1.44	1.41	1.18	1.18	1.19	1.16	1.06	0.372

Furthermore, the analysis revealed a correlation between maternal age and infant mortality rates. Infants born to younger mothers, particularly those in their teenage years (under 15 years), displayed higher mortality rates (6.82 per 1,000 infants; p-value=0.374), followed by the 45-49 years age group (5.42 per 1,000 infants; p-value=0.152). Conversely, infants born to mothers in their mid-20s to early 30s tended to experience lower mortality rates. The impact of maternal education on infant mortality rates was also evident. Infants born to mothers with higher levels of education exhibited lower mortality rates, indicating a positive relationship between maternal education and child health outcomes. This suggests the importance of education as a determinant of maternal and child well-being.

Trends in infant mortality rates based on geographical variables

**Figure 2:**
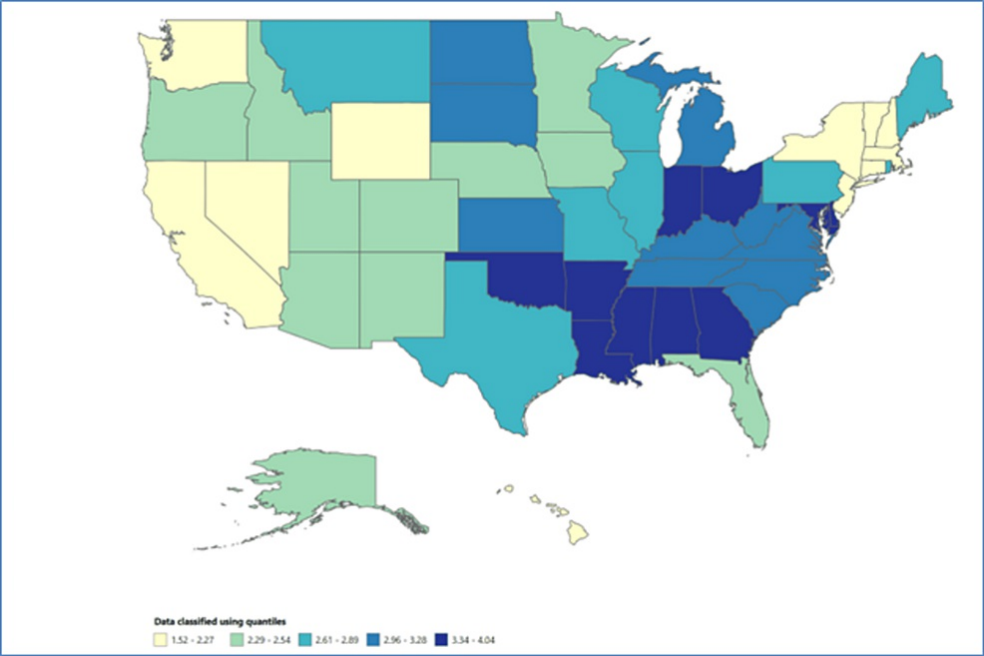
Mortality rate across the United States in 2020

The examination of trends in infant mortality rates took into consideration geographic variables, including state, census division, and HHS region, as presented in Table 4. Variation in infant mortality rates was evident across different states. Mississippi exhibited the highest rate in 2020 (4.04 per 1,000 infants), followed by Louisiana (3.94 per 1,000 infants). The analysis by the census division highlighted distinct trends in infant mortality rates across different divisions. Division 3: East North Central demonstrated the highest mortality rates (3.18 per 1,000 infants), while others experienced relatively lower rates (Figure 2). Examination of infant mortality rates by HHS regions revealed disparities in rates across the different regions. HHS regions 3 and 5 recorded the highest mortality rates (3.08 per 1,000 infants), while others experienced relatively lower rates. This variation underscores the importance of geographic location-based interventions and healthcare strategies to address local disparities in infant mortality rates.

**Table 4:** Mortality rate trend based on location (2007-2020) *The top 5 selected states with the highest mortality rate are presented in the table HHS: health and human services, MMWR: morbidity and mortality weekly report, AL: Alabama, AK: Alaska, AZ: Arizona, AR: Arkansas, CA: California, CO: Colorado, CT: Connecticut, DE: Delaware, FL: Florida, GA: Georgia, HI: Hawaii, ID: Idaho, IL: Illinois, IN: Indiana, IA: Iowa, KS: Kansas, KY: Kentucky, LA: Louisiana, ME: Maine, MD: Maryland, MA: Massachusetts, MI: Michigan, MN: Minnesota, MS: Mississippi, MO: Missouri, MT: Montana, NE: Nebraska, NV: Nevada, NH: New Hampshire, NJ: New Jersey, NM: New Mexico, NY: New York, NC: North Carolina, ND: North Dakota, OH: Ohio, OK: Oklahoma, OR: Oregon, PA: Pennsylvania, RI: Rhode Island, SC: South Carolina, SD: South Dakota, TN: Tennessee, TX: Texas, UT: Utah, VT: Vermont, VA: Virginia, WA: Washington, WV: West Virginia, WI: Wisconsin, WY: Wyoming

Variables		Number of deaths	Mortality rate per 1,000
Census division	Division 1: New England	4,475	2.12
	Division 2: Middle Atlantic	15,027	2.22
	Division 3: East North Central	25,003	3.18
	Division 4: West North Central	10,259	2.71
	Division 5: South Atlantic	31,808	3.03
	Division 6: East South Central	11,240	3.4
	Division 7: West South Central	22,485	2.96
	Division 8: Mountain	10,251	2.38
	Division 9: Pacific	19,323	2.1
HHS region	HHS Region #1 CT, ME, MA, NH, RI, VT	4,475	2.12
	HHS Region #2 NJ, NY	9,333	1.95
	HHS Region #3 DE, DC, MD, PA, VA, WV	15,392	3.08
	HHS Region #4 AL, FL, GA, KY, MS, NC, SC, TN	33,350	3.09
	HHS Region #5 IL, IN, MI, MN, OH, WI	27,216	3.08
	HHS Region #6 AR, LA, NM, OK, TX	23,400	2.94
	HHS Region #7 IA, KS, MO, NE	7,090	2.82
	HHS Region #8 CO, MT, ND, SD, UT, WY	5,485	2.48
	HHS Region #9 AZ, CA, HI, NV	18,748	2.1
	HHS Region #10 AK, ID, OR, WA	5,382	2.31
State*	Mississippi	2,235	4.04
	Louisiana	3,447	3.94
	Arkansas	2,110	3.93
	Oklahoma	2,679	3.66
	Alabama	3,043	3.63

## Discussion

Examining trends and factors linked to infant mortality rates, specifically tied to the primary causes of death, has yielded crucial insights into the intricate interplay of determinants influencing infant well-being and survival. These insights hold implications for healthcare interventions, policy shaping, and the trajectory of future research. The distinctive trends observed for each leading cause illuminate the multifaceted landscape of infant mortality. Congenital malformations, deformations, and chromosomal abnormalities (total number of deaths: 68,619; mortality rate: 1.24 per 1,000 infants) emerged as significant contributors, with rates fluctuating over the study period. This was followed by short gestation and low birth weight (total number of deaths: 57,078; mortality rate: 1.03 per 1,000 infants), and a consistent downward trend in disorders reflects the impact of improved maternal and prenatal care, promoting healthier birth outcomes. SIDS was the third most common cause of infant mortality (total number of deaths: 24,175; mortality rate: 0.43 per 1,000 infants) and demonstrated fluctuations, highlighting the ongoing challenges in understanding its intricate etiology and developing preventive strategies. These downward trends in infant mortality and the leading causes are consistent with previously published reports on infant mortality in the United States [5,17]. While overall rates were similar, a slightly higher mortality rate was observed among male infants compared to female infants. This finding aligns with a previously published National Vital Statistics Reports suggesting that male infants might be more susceptible to certain health complications and congenital conditions [13,15-16].

The analysis of infant mortality rates across different racial categories highlights significant disparities in infant health outcomes. Black infants exhibited the highest mortality rates, followed by American Indian or Alaska Native, White, and Asian infants. Similar trends were also observed in previously published studies in the United States [17-20]. These findings draw attention to the importance of addressing racial disparities in healthcare access, socio-economic conditions, and systemic factors that contribute to differential infant mortality rates among racial groups. The consistently observed higher mortality rates during the neonatal period (1-23 hours) and the subsequent 28-364 days period underscore the critical need for intensive healthcare interventions and support for newborns during these early stages of life, which is consistent with findings from other studies as well [5,18].

These findings collectively underscore the intricate relationship between maternal variables and infant mortality rates. The higher mortality rates among infants born to teenage mothers and the 45-49 years age group emphasize the vulnerability of these populations. Similar results were reported by Singh et al. in a pooled analysis of data over the period from 1915 to 2017 in a study conducted in the United States [8,20]. Teenage pregnancies have been linked to inadequate prenatal care and increased health risks, potentially contributing to higher infant mortality rates. Similarly, advanced maternal age presents its challenges, including increased risks of complications during pregnancy and childbirth that could impact infant health outcomes [5]. Infants born to mothers with higher levels of education exhibited lower mortality rates, indicating a positive relationship between maternal knowledge, healthcare access, and child health outcomes [17-22]. Our study's findings indicated that infants born through vaginal delivery displayed slightly lower mortality rates compared to those delivered via cesarean section. Similar trends were reported by Holmes et al. in an epidemiological study comparing the delivery methods of vaginal and cesarean section [23]. This association might reflect differences in postnatal care practices, recovery rates, and potential health implications associated with cesarean deliveries [13-17,24]. Strategies aimed at improving maternal education, enhancing healthcare access during pregnancy and childbirth, and promoting informed choices regarding delivery methods can potentially contribute to the reduction of infant mortality rates and overall improvement in child health. This study underscores the significance of geographical variables in shaping trends in infant mortality rates. The observed disparities across states, census divisions, and HHS regions emphasize the need for targeted interventions that address region-specific challenges and promote equitable access to quality healthcare. The findings of this study were found to be consistent with previously published studies [13-19,25-27].

Despite its valuable insights, the study has certain limitations that warrant consideration. Firstly, the analysis relies on data accuracy and completeness within the CDC WONDER database. Variations in reporting practices or misclassification of causes of death could potentially introduce bias into the findings. Additionally, the retrospective nature of the study restricts the establishment of causal relationships between factors and infant mortality rates. Unmeasured confounding variables could influence the observed associations, highlighting the need for cautious interpretation. The generalizability of the study's findings might be limited to populations and contexts covered by the CDC WONDER database. Moreover, temporal changes in healthcare practices and societal factors occurring before or after the study period might not be fully captured, potentially impacting the accuracy of trend analysis.

This study also possesses several strengths that contribute to its significance. Utilizing the comprehensive CDC WONDER database provides access to a substantial pool of infant mortality cases, enhancing the statistical power of the analysis. The consideration of a wide array of variables, including demographic, maternal, and geographical factors, facilitates a nuanced understanding of the complex interactions influencing infant mortality rates. The study's longitudinal approach, spanning from 2007 to 2020, enables the identification of temporal trends and changes in mortality rates over time. Furthermore, the study's findings have practical implications for public health policies and interventions, aiding in the development of evidence-based strategies to improve infant health outcomes. Overall, the study adds to the existing knowledge base on infant mortality, serving as a valuable resource for researchers, policymakers, and healthcare practitioners.

## Conclusions

In summary, the analysis of infant mortality trends based on the top three leading causes of death from 2007 to 2020, using data from the CDC WONDER database, revealed an overall mortality rate of 2.69 deaths per 1,000 individuals. Noteworthy variations were observed across causes, with congenital malformations, deformations, and chromosomal abnormalities showing fluctuating rates, disorders related to short gestation and low birth weight displaying a declining pattern, and SIDS exhibiting fluctuations. This research underscores the significance of maternal age (30-34 years and 35-39 years), race/ethnicity (Black or African American and White), birthplace (in hospital), and maternal education (master's degree) as factors influencing mortality rates. These findings provide valuable guidance for healthcare policies and interventions aimed at reducing infant mortality rates. This emphasizes the need for tailored interventions and strategies to address the multifaceted influences on infant health and survival.
